# Persistent Severe Acute Kidney Injury Among Critically Ill Patients: Outcomes and Predictive Markers—A Single‐Center Retrospective Cohort Study

**DOI:** 10.1155/ccrp/6920702

**Published:** 2026-02-17

**Authors:** Tung Phi Nguyen, Thang Trong Khong, Hoai Thi Thu Vu, Nam Ngoc Phuong Nguyen, Phong Van Phan, Hue Thi Le, Tra Thi Hoang, Huyen Thi Nguyen, Loan Thi Phan, Yen Thi Kim Nguyen, Phuong Khanh Nguyen Hoang

**Affiliations:** ^1^ Department of Intensive Care Unit, Vinmec International Hospital, Vinmec Healthcare System, Ho Chi Minh City, Vietnam; ^2^ College of Health Sciences, VinUniversity, Hanoi, Vietnam; ^3^ Pharmacy Department, Vinmec International Hospital, Vinmec Healthcare System, Ho Chi Minh City, Vietnam

**Keywords:** acute kidney injury, critical illness, intensive care units, neutrophil-to-lymphocyte ratio, persistent acute kidney injury, persistent severe acute kidney injury, renal replacement therapy, systemic immune-inflammation index

## Abstract

**Background:**

Persistent severe acute kidney injury (PS‐AKI)—recently standardized as Kidney Disease: Improving Global Outcomes (KDIGO) Stage 3 persisting ≥ 72 h, or renal replacement therapy/death after Stage 3 diagnosis—has emerged as a trajectory‐based phenotype complementing conventional KDIGO staging. Evidence in contemporary intensive care unit (ICU) cohorts remains limited.

**Methods:**

We retrospectively studied adults admitted to a tertiary ICU (January 2024–June 2025). Acute kidney injury (AKI) was staged per KDIGO 2012, with trajectories classified as Stage 1 AKI, transient AKI (Stage 2‐3 resolving within 48 h), persistent mild–moderate AKI, or PS‐AKI. The primary outcome was in‐hospital mortality; secondary outcomes included renal recovery. Predictors of PS‐AKI were explored using logistic regression and gradient boosting with SHAP attribution.

**Results:**

Among 139 ICU patients with AKI screened, 106 met criteria. Most AKI was community‐acquired (97/106, 91.5%). PS‐AKI accounted for 23% (24/106) and carried the worst outcomes, with in‐hospital mortality 54% and renal recovery 17%. Within Stage 2‐3, PS‐AKI was associated with substantially worse outcomes than non‐PS trajectories (mortality 54% vs 10.8%; adjusted HR for death 2.23, 95% CI 0.69–7.21; adjusted OR for renal recovery 0.07, 95% CI 0.01–0.24). A 72‐h landmark analysis showed similar but nonsignificant trends. Inflammatory profiles distinguished PS‐AKI, with higher neutrophil‐to‐lymphocyte ratio (NLR), C‐reactive protein (CRP), and lower platelets. The composite NLR‐to‐platelet ratio (NLR/PLT) was independently associated with PS‐AKI (adjusted OR 2.51 per doubling, 95% CI 1.52–4.12; AUC 0.86), while the systemic immune‐inflammation index (SII) showed no significant association.

**Conclusions:**

In this predominant community‐acquired ICU cohort, PS‐AKI was common and strongly associated with poor in‐hospital outcomes. The co‐occurrence of inflammation and thrombocytopenia, summarized by NLR/PLT, may represent a simple exploratory signal for early‐risk appraisal. These findings support further research into trajectory‐based AKI phenotypes and the potential utility of inflammation–hematologic markers in predicting persistence.

## 1. Background

Acute kidney injury (AKI) is common in critically ill patients and is associated with excess morbidity and mortality [1–4]. The Kidney Disease: Improving Global Outcomes (KDIGO) staging system provides a point‐in‐time assessment of severity [5], but outcomes depend not only on the initial stage but also on subsequent trajectories of recovery or persistence [2, 6, 7]. Recognizing this, the 16th Acute Disease Quality Initiative (ADQI 16) emphasized the importance of persistent AKI as a modifier of risk [6].

Building on this concept, the RUBY study prospectively enrolled patients with KDIGO Stage 2‐3 and used a composite endpoint capturing persistence of severe injury—KDIGO Stage 3 lasting ≥ 72 h, or renal replacement therapy (RRT) or death after reaching Stage 3—demonstrating substantially worse kidney and survival outcomes [8]. In 2025, Gómez et al. standardized a related definition of persistent severe AKI (PS‐AKI)—persistence of KDIGO Stage 3 for ≥ 72 h, RRT after Stage 3, or death following stage‐3 diagnosis—and confirmed its adverse prognostic significance in a large multicenter intensive care unit (ICU) cohort [2]. Given the recency of this definition, evidence on PS‐AKI remains limited.

To address this gap, we conducted a single‐center retrospective cohort study to classify AKI trajectories using the Gómez definition of PS‐AKI, evaluate their association with in‐hospital outcomes, and explore simple clinical and laboratory predictors.

## 2. Materials and Methods

### 2.1. Study Design and Population

We conducted a retrospective cohort study at a tertiary academic hospital in Vietnam affiliated with VinUniversity. All consecutive adults (≥ 18 years) admitted between January 2024 and June 2025 who stayed ≥ 24 h were screened. Eligible patients had AKI at ICU admission or developed AKI during the ICU stay according to KDIGO 2012 criteria. Exclusion criteria were (i) age < 18 years; (ii) end‐stage kidney disease (ESKD) or prior kidney transplantation; (iii) admission serum creatinine (sCr) ≥ 353.6 μmol/L (4.0 mg/dL) or estimated glomerular filtration rate (eGFR) ≤ 15 mL/min/1.73 m^2^ without supporting evidence to exclude ESKD (defined as either prior outpatient creatinine confirming AKI or recovery of kidney function at discharge); and (iv) insufficient sCr/urine data to adjudicate AKI. The protocol was approved by the Institutional Review Board of VinUniversity with a waiver of informed consent.

### 2.2. Study Size

All consecutive eligible admissions within the study window were included. No a priori sample‐size calculation was performed because the 2025 standardized PS‐AKI definition lacked context‐appropriate incidence or effect‐size data; using estimates from large multicenter United States cohorts with different case‐mix risked misspecification. We therefore prioritized comprehensive accrual and reporting of precision (95% CIs) over formal power targeting.

### 2.3. AKI Definition and Trajectories

AKI was defined and staged per KDIGO 2012 using changes in sCr and/or urine output [5]. Reference creatinine was determined hierarchically: (i) mean of outpatient values 7–365 days preadmission (or single value if only one, used in 45/106 patients, 42.5%) [9, 10]; (ii) if no outpatient values and no chronic kidney disease (CKD), the lower of the sCr back‐calculated from an eGFR of 75 mL/min/1.73 m^2^ (Modification of Diet in Renal Disease [MDRD] equation) or the in‐hospital nadir (56/106, 52.8%) [5, 11]; and (iii) if known CKD and no outpatient values, the in‐hospital nadir (5/106, 4.7%) [5]. This approach follows KDIGO guidance and aimed to mitigate misclassification in a predominantly community‐acquired AKI cohort [5, 11]. Community‐acquired AKI was assigned when any of the following were present: (1) sCr was elevated at ICU admission and declined during the hospital stay; (2) sCr was elevated at ICU admission and remained elevated or increased, with preadmission measurements documenting that AKI had started before admission; or (3) kidney function was normal at ICU admission but AKI criteria were fulfilled within 48 h, together with documentation that precipitating factors had been present before admission. All remaining cases were labeled hospital‐acquired AKI [12].

Patients were classified into four trajectories: (1) Stage 1 AKI—maximum KDIGO Stage 1 during the ICU stay; among patients whose maximum KDIGO stage was ≥ 2, we further defined three mutually exclusive subgroups: (2) transient AKI—KDIGO Stage 2‐3 with recovery to no AKI within 48 h of first diagnosis; (3) persistent mild–moderate AKI—sustained KDIGO Stage 2‐3 not meeting persistent severe criteria and not transient; and (4) PS‐AKI—after reaching KDIGO Stage 3, persistence ≥ 72 h, initiation of RRT, or death. For analytic purposes, transient AKI and persistent mild–moderate AKI were collectively referred to as nonpersistent severe AKI. This classification follows the RUBY framework and the standardized definition proposed by Gómez et al. [2].

## 3. Outcomes

The primary outcome was in‐hospital mortality across AKI trajectories, with a prespecified contrast of PS‐AKI versus other groups. Secondary outcomes included renal recovery (assessed at hospital discharge, defined as discharge sCr ≤ 150% of reference and no dialysis dependence) [2, 7], ICU and hospital length of stay, and RRT dependence at discharge. Exploratory analyses evaluated predictors of PS‐AKI.

### 3.1. Data Collection

We abstracted demographics; comorbidities (including CKD and Charlson Comorbidity Index [CCI]); illness severity (Acute Physiology and Chronic Health Evaluation II [APACHE II], and Sequential Organ Failure Assessment [SOFA], including the cardiovascular and renal SOFA component); and pre‐AKI inflammatory/hemodynamic markers and medication exposures. Nephrotoxic and diuretic agents were identified from medication/administration records and coded as binary (any use vs none); the full list of screened agents is provided in Supporting Table S1. Data were collected from ICU admission up to—but not including—the calendar day of AKI diagnosis; for patients presenting with AKI on the admission day, exposures and markers were abstracted from that admission day. Within this window, we recorded the lowest platelet count (PLTmin) and the highest values of neutrophil‐to‐lymphocyte ratio (NLR), NLR‐to‐platelet ratio (NLR/PLT, calculated as NLR ÷ PLT × 100), systemic immune‐inflammation index (SII; neutrophils × platelets ÷ lymphocytes), C‐reactive protein (CRP), lactate, procalcitonin, and bilirubin.

### 3.2. Statistical Analysis

Continuous variables are reported as median [IQR] and categorical as *n* (%). Group comparisons used Kruskal–Wallis or Fisher’s exact tests. Survival across trajectories was assessed with Kaplan–Meier curves and log‐rank tests. Associations of PS‐AKI with in‐hospital mortality were estimated with Cox proportional hazards models adjusted for nonrenal SOFA and with renal recovery by logistic regression. Predictors of PS‐AKI were explored using (i) a prespecified two‐variable logistic model and (ii) gradient boosting (XGBoost) with SHapley additive explanations (SHAP) attribution. Sensitivity analyses included risk‐set landmarking and exclusion of early deaths (details in Supporting Methods) and restriction to patients with outpatient baseline sCr. Skewed biomarkers (e.g., NLR, NLR/PLT, CRP, SII, lactate) were analyzed on the log_2_ scale (per doubling). Analyses were performed in *R* (two‐sided *α* = 0.05). Missing data policy: we conducted complete‐case analyses for each model and did not perform imputation. Descriptive tables report available‐case denominators, and footnotes indicate the number of observations used for variables with missingness.

## 4. Results

### 4.1. Study Population and AKI Trajectories

**FIGURE 1:**
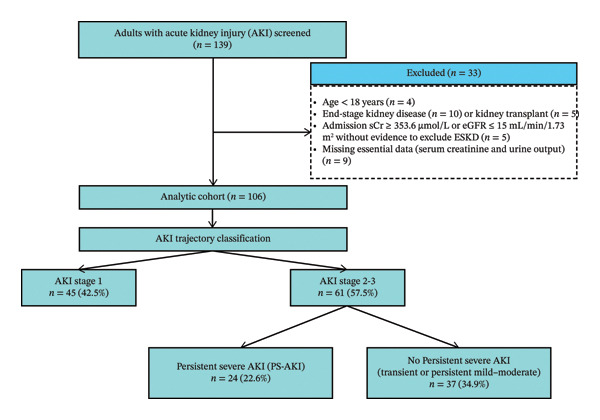
Flow diagram of study population and AKI trajectory classification.

Among 139 ICU patients with AKI screened, 106 met eligibility criteria (Figure 1). AKI was already present at ICU admission in 93/106 patients and was diagnosed within 48 h of admission in a further 4/106, so that 97/106 (91.5%) fulfilled criteria for community‐acquired AKI. Of these, 45 (42%) were classified as Stage 1 AKI, 23 (22%) as transient AKI, 14 (13%) as persistent mild–moderate AKI, and 24 (23%) as PS‐AKI. Among these, 10 met the time‐based criterion (Stage 3 persisting ≥ 72 h), 3 died after reaching Stage 3, and 11 initiated RRT following Stage 3.

### 4.2. Baseline Characteristics and Outcomes

Baseline characteristics and outcomes across trajectories are summarized in Table 1. Severity increased stepwise, with higher SOFA and cardiovascular SOFA and APACHE II scores in PS‐AKI. Inflammatory profiles showed marked differences: PS‐AKI patients had lower platelet counts together with higher NLR, NLR/PLT, and CRP, while procalcitonin did not differ significantly. ICU and hospital length of stay were longest in PS‐AKI, corresponding to the lowest renal recovery (17%) and the highest in‐hospital mortality (54%).

**TABLE 1:** Baseline characteristics and outcomes across AKI trajectories.

Characteristic	All (*N* = 106)	AKI Stage 1 (*n* = 45)	Transient AKI (*n* = 23)	Persistent mild–moderate (*n* = 14)	Persistent severe (*n* = 24)	*p* value
Demographics and comorbidity						
Age, years	68.5 (55.0–85.0)	69.0 (56.0–82.0)	55.0 (37.0–87.0)	70.0 (62.0–83.0)	74.0 (59.0–86.0)	0.132
Female sex, *n* (%)	35 (33)	16 (36)	3 (13)	7 (50)	9 (38)	0.066
Sepsis, *n* (%)	54 (51)	22 (49)	9 (39)	7 (50)	16 (67)	0.301
CKD, *n* (%)	17 (16)	6 (13)	1 (4)	2 (14)	8 (33)	0.060
CCI (points)	5.0 (3.0–6.0)	4.0 (2.0–5.0)	3.0 (0.0–5.0)	6.0 (4.0–7.0)	6.0 (4.0–8.0)	< 0.001
BMI (kg/m^2^)	23.5 (20.4–26.8)	23.9 (20.8–27.6)	23.6 (21.7–27.5)	23.5 (20.6–26.6)	20.9 (19.3–25.8)	0.300
Baseline creatinine (μmol/L)	79.3 (64.5–90.9)	80.0 (66.0–88.0)	71.0 (62.0–80.0)	76.0 (57.0–111.0)	89.0 (73.0–115.0)	0.025
Baseline severity						
First AKI stage–Stage 1, *n* (%)	50 (47)	45 (100)	2 (9)	3 (21)	0 (0)	< 0.001
Stage 2, *n* (%)	38 (36)	0 (0)	18 (78)	9 (64)	11 (46)	
Stage 3, *n* (%)	18 (17)	0 (0)	3 (13)	2 (14)	13 (54)	
SOFA admission (points)	5 (4–7)	4 (3–6)	5 (4–8)	6 (4–6)	7 (6–11)	< 0.001
Cardiovascular SOFA admission (points)	0 (0–3)	0 (0–1)	0 (0–3)	0 (0–3)	3 (1–4)	< 0.001
APACHE II score (points)	24 (19–28)	18 (16–19)	25 (22–27)	28 (27–30)	29 (28–32)	< 0.001
Pre‐AKI inflammatory and hemodynamic markers						
PLT min, × 10^9^/L	177.6 (118.0–227.1)	190.0 (143.0–229.0)	196.0 (127.0–214.0)	234.0 (176.0–284.0)	99.0 (61.0–153.0)	< 0.001
NLR max	11.0 (6.3–25.3)	8.0 (5.0–16.0)	11.0 (6.0–18.0)	11.0 (8.0–14.0)	27.0 (15.0–43.0)	< 0.001
NLR/PLT max, × 100 per 10^9^/L	6.3 (2.8–19.8)	5.0 (2.0–13.0)	5.0 (3.0–14.0)	5.0 (2.0–6.0)	26.0 (13.0–43.0)	< 0.001
SII max, × 10^9^/L	1829 (910–3153)	1420 (630–2790)	1851 (708–3163)	2230 (1194–3481)	2423 (1348–4967)	0.150
Procalcitonin max (ng/mL)	2.3 (0.4–13.4)	2.0 (0.0–13.0)	2.0 (0.0–18.0)	2.0 (0.0–8.0)	3.0 (1.0–19.0)	0.464
CRP max (mg/L)	91.9 (18.1–177.4)	43.0 (15.0–155.0)	99.0 (14.0–146.0)	32.0 (10.0–152.0)	162.0 (118.0–289.0)	0.001
Lactate max (mmol/L)	1.7 (1.0–3.1)	1.5 (1.0–2.3)	1.7 (0.8–3.1)	2.1 (1.5–3.3)	3.1 (1.3–6.6)	0.026
Bilirubin max (μmol/L)	15.0 (11.4–27.7)	14.0 (11.0–18.0)	17.0 (12.0–34.0)	14.0 (11.0–26.0)	31.0 (14.0–94.0)	0.030
Exposures (pre‐AKI)						
Any diuretic before AKI, *n* (%)	78 (74)	30 (67)	15 (65)	13 (93)	20 (87)	0.078
Any nephrotoxin before AKI, *n* (%)	16 (15)	6 (13)	1 (5)	2 (14)	7 (30)	0.106
Outcomes						
ICU stay, days	7.0 (4.0–14.0)	5.0 (4.0–9.0)	6.0 (4.0–9.0)	10.0 (7.0–18.0)	14.0 (7.0–33.0)	< 0.001
Hospital stay, days	11.5 (8.0–19.0)	10.0 (7.0–17.0)	9.0 (8.0–13.0)	19.0 (13.0–20.0)	20.0 (13.0–32.0)	0.001
Renal recovery, *n* (%)	70 (66)	38 (84)	21 (91)	7 (50)	4 (17)	< 0.001
RRT dependence at discharge, *n* (%)	2 (2)	0 (0)	0 (0)	0 (0)	2 (8)	0.028
Death, *n* (%)	18 (17)	1 (2)	0 (0)	4 (29)	13 (54)	< 0.001

*Note:* Values are median (IQR) or *n* (%). *p*‐values by Kruskal–Wallis (continuous) or Fisher’s exact (categorical). Procalcitonin values were available for 84/106 patients (missing in 18 Stage 1; 3 transient; and 1 persistent mild–moderate AKI cases). Data on fluid balance prior to AKI onset were sparsely and inconsistently recorded (available in a small minority of patients) and were therefore not summarized or included in analyses. In addition, detailed hemodynamic profiles (MAP and vasopressor requirements) at admission are provided in Supporting Table S15.

### 4.3. Survival Across Trajectories

**FIGURE 2:**
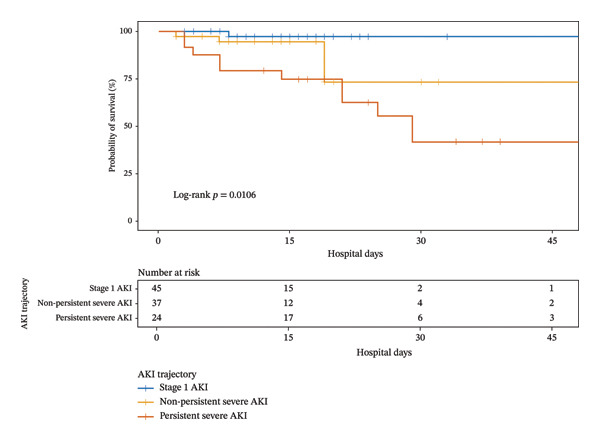
Kaplan–Meier survival across AKI trajectories.

Kaplan–Meier curves (Figure 2) demonstrated significant survival differences (global log‐rank *p* = 0.0106), with PS‐AKI showing the poorest survival and Stage 1 AKI the best. Pairwise comparisons and extended numbers‐at‐risk are provided in Supporting Table S2.

### 4.4. Effect of PS‐AKI Within KDIGO Stage 2‐3

Restricting analyses to KDIGO Stage 2‐3 patients, outcomes were substantially worse in PS‐AKI compared with nonpersistent severe AKI (Table 2). Mortality was 54.0% vs 10.8%, and renal recovery 16.7% vs 87.6%. In Cox models, PS‐AKI showed elevated hazard ratios for in‐hospital mortality (unadjusted HR 2.59, 95% CI 0.80–8.44; adjusted HR 2.23, 95% CI 0.69–7.21), though confidence intervals were wide. Associations with renal recovery were strong (adjusted OR 0.07, 95% CI 0.01–0.24; *p* < 0.001). These findings were directionally consistent across single‐covariate adjustments (age, CCI, SOFA at admission, APACHE II; Supporting Tables S3‐S4) and in the full‐cohort analysis (Supporting Table S5). To address immortal‐time bias, similar associations were observed when excluding early deaths within 72 h (Supporting Table S6) and in a prespecified 72 h landmark analysis (*n* = 50; postlandmark deaths 9/20 vs 1/30; aHR 4.13, 95% CI 0.48–35.2; *p* = 0.19; Supporting Figure S1 and Table S7). Descriptive patterns of early, late, and never reversal across KDIGO Stage 2‐3 trajectories are reported in Supporting Table S14.

**TABLE 2:** Association of PS‐AKI with in‐hospital mortality and renal recovery within KDIGO Stage 2‐3.

Outcome	Nonpersistent severe AKI	Persistent severe AKI	*p* value	Unadjusted HR/OR (95% CI), *p*	Adjusted^∗^HR/OR (95% CI), *p*
In‐hospital mortality, n/N (%)	4/37 (10.8)	13/24 (54.0)	< 0.01	2.59 (0.80–8.44), 0.114	2.23 (0.69–7.21), 0.181
Renal recovery, n/N (%)	28/37 (87.6)	4/24 (16.7)	< 0.01	0.06 (0.02–0.22), < 0.001	0.07 (0.01–0.24), < 0.001

^∗^Adjusted for nonrenal SOFA within 24 h of ICU admission. Time origin for survival was ICU admission; patients were censored at hospital discharge.

### 4.5. Predictors of PS‐AKI

Within KDIGO Stage 2‐3, univariable analysis identified several factors significantly associated with PS‐AKI (all *p* < 0.05), including higher NLR/PLT, higher NLR, initial AKI Stage 3 (vs 2), lower platelet count, higher APACHE II, higher CRP, and higher CCI (all *p* < 0.05; Table 3). SOFA at admission and CKD showed only nominal significance, while SII and procalcitonin were not associated. The full list of univariable estimates, including variables not reaching significance, is provided in Supporting Table S8. In the prespecified two‐variable model (age + NLR/PLT), the ratio remained independently associated with PS‐AKI (adjusted OR 2.51 per doubling, 95% CI 1.52–4.12), achieving good discrimination (AUC 0.86, 95% CI 0.76–0.96). Alternative two‐variable models substituting NLR, platelet count, or CRP yielded comparable discrimination (AUC ≈ 0.80–0.88; Supporting Table S9). Model performance metrics, including cross‐validation, calibration, and decision thresholds, are reported in the Supplement (TRIPOD assessment; Supporting Methods/Results for Tables S9‐S10).

**TABLE 3:** Univariable and multivariable predictors of persistent severe AKI (KDIGO Stage 2‐3).

Predictor	OR (95% CI)	*p*‐value
Univariable		
NLR/PLT (per doubling)	2.50 (1.53–4.06)	< 0.001
NLR max (per doubling)	2.89 (1.57–5.32)	< 0.001
First AKI stage (3 vs 2)	7.30 (2.26–23.58)	< 0.001
Platelet count min (per 10 × 10^9^/L)	0.88 (0.82–0.96)	0.002
APACHE II (per 5 points)	2.97 (1.44–6.13)	0.003
CRP max (per doubling)	1.79 (1.20–2.68)	0.004
Charlson comorbidity index	1.33 (1.08–1.64)	0.008
SOFA at admission, points	1.20 (1.03–1.39)	0.020
CKD (yes vs no)	5.67 (1.32–24.25)	0.020
Any nephrotoxin before AKI (yes vs no)	4.67 (1.07–20.35)	0.040
Multivariable (primary model)		
Age + NLR/PLT: aOR for NLR/PLT (per doubling)	2.51 (1.52–4.12)	< 0.001
Model performance (AUC)	0.86 (95% CI 0.76–0.96)	—

*Note:* Continuous predictors were log_2_‐transformed where modeled “per doubling” (e.g., NLR/PLT, NLR, CRP). The primary multivariable model was limited to two prespecified clinical variables to mitigate overfitting. AUC, area under the ROC curve.

Abbreviations: aOR, adjusted odds ratio; CRP, C‐reactive protein; NLR, neutrophil‐to‐lymphocyte ratio; PLT, platelet count.

### 4.6. Model Explanation

**FIGURE 3:**
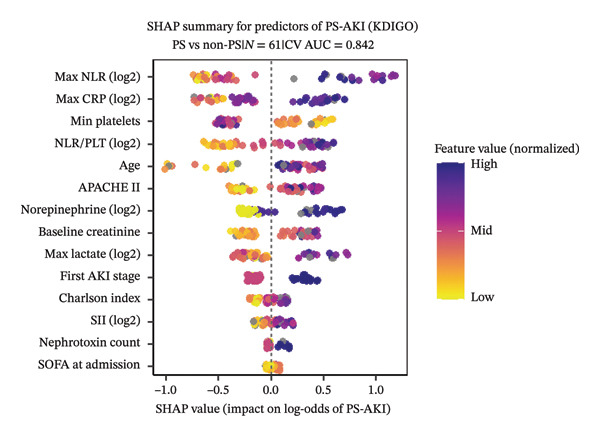
SHAP summary plot for predictors of PS‐AKI (KDIGO Stage 2‐3).

Exploratory gradient‐boosted models with SHAP corroborated these findings, with NLR/PLT, initial AKI stage, platelet count, and CRP among the most influential features (Figure 3). Median absolute SHAP rankings for the Stage 2‐3 subset and the full cohort are presented in Supporting Tables S11‐S12, with the full‐cohort bee‐swarm shown in Supporting Figure S2.

Features are ordered by median absolute SHAP value, highlighting NLR/PLT, initial AKI stage, platelet count, and CRP as the most influential predictors. Complementary analysis for the entire cohort is shown in Supporting Figure S2.

## 5. Discussion

This single‐center ICU study applied the 2025 definition of PS‐AKI proposed by Gómez et al. to quantify its occurrence, outcomes, and exploratory predictors in a predominantly community‐acquired cohort.

### 5.1. Key Findings in Context of Prior Work

PS‐AKI accounted for 23% of all AKI (24/106) and carried the worst prognosis, with in‐hospital mortality of 54% and renal recovery of 17%. Within KDIGO Stage 2‐3, PS‐AKI was associated with higher mortality risk and substantially lower renal recovery than nonpersistent severe AKI, with directionally consistent findings in sensitivity analyses (Supporting Table S5). These observations align with multicenter reports [2]. In Gómez et al. (2025), 12.4% of patients with Stage 2‐3 AKI progressed to PS‐AKI, which was associated with a higher 90‐day mortality and markedly reduced renal recovery (OR ≈ 0.14). Earlier, the RUBY study (Hoste et al., 2020) prospectively enrolled ICU patients with KDIGO Stage 2‐3 and used a composite endpoint—Stage 3 persisting ≥ 72 h, or Stage 3 followed by RRT or death—with approximately one‐third meeting this endpoint and experiencing significantly higher mortality and 90‐day adverse kidney events [8]. Compared with these cohorts, our study found a higher proportion of PS‐AKI among Stage 2‐3 cases, while the effect on renal recovery was similar in direction and magnitude, with wide confidence intervals around mortality reflecting limited sample size. The case‐mix in our ICU—where more than 90% of AKI was community‐acquired—likely contributed to this difference, as many patients already met KDIGO Stage 2‐3 at the time of first ICU assessment. Together, these findings support PS‐AKI as a clinically relevant trajectory across settings, including predominantly community‐acquired AKI populations such as ours.

## 6. Methodological Considerations and Case‐Mix

Because most AKI in our setting was community‐acquired, we applied a hierarchical baseline‐creatinine strategy (outpatient values when available; otherwise MDRD‐75 vs in‐hospital nadir) to minimize misclassification, consistent with KDIGO guidance [5, 13]. Notably, no PS‐AKI patient in our cohort had Stage 1 as the initial stage, likely reflecting later presentation from the community, where patients were already at Stage 2‐3 upon first assessment. In contrast, nosocomial AKI more often allows observation of progression from Stage 1 to persistence. Regarding the clinical course of KDIGO Stage 2‐3, mortality was not only driven by PS‐AKI but also driven by the persistent mild–moderate trajectory, which showed higher in‐hospital mortality than transient AKI (29% vs 0%). Prior work has highlighted that recovery phenotypes—early reversal, late reversal, and nonrecovery—are strongly linked to outcomes and informed the ADQI framework for persistent AKI [7]. In our cohort, transient AKI consisted entirely of early reversal, whereas PS‐AKI was dominated by never‐reversal and the persistent mild–moderate group combined late and never‐reversal (Supporting Table S14). Although purely descriptive, this pattern offers a plausible explanation for the intermediate but clinically relevant mortality in persistent mild–moderate AKI and is consistent with the concept that both the depth and duration of kidney injury influence prognosis.

### 6.1. Inflammation, Thrombocytopenia, and PS‐AKI (Exploratory Signal)

Within KDIGO Stage 2‐3, PS‐AKI in our cohort clustered with higher systemic inflammatory markers and thrombocytopenia. The PS‐AKI group had markedly higher NLR and CRP together with lower platelet counts; the median NLR was 27.0 (15.0–43.0), a range that aligns with severe‐to‐critical inflammatory stress on the NLR‐meter scale. These features align with an inflammatory–endothelial mechanism, in which systemic inflammation and glycocalyx injury disturb microvascular flow and renal microcirculatory dysfunction may persist despite apparently corrected macrohemodynamics [14]. Experimental and translational studies implicate the angiopoietin‐2/Tie‐2 axis and related endothelial biomarkers in endothelial destabilization, thromboinflammation, and organ injury, while chronic inflammatory tone has also been linked to AKI development and long‐term mortality [14–18]. Observational data across ICU settings complement these mechanistic insights and consistently link thrombocytopenia with adverse renal outcomes. In sepsis, declining platelet counts are associated with multiple organ failure, AKI, and mortality [19, 20]; low or falling platelets in patients receiving CRRT predict worse survival [21, 22]; and nadir platelet counts within the first 48 h can signal subsequent AKI [23]. Persistent AKI, compared with transient AKI, has also been associated with more pronounced inflammation, coagulation abnormalities, and endothelial injury [24]. Importantly, in a large ICU network, lower minimum platelet count before AKI independently predicted PS‐AKI [2], reinforcing the biological and prognostic relevance of thrombocytopenia in this phenotype. Our findings are directionally consistent with this literature but exploratory. Platelet counts were lowest in PS‐AKI (median ≈ 99 × 10^9^/L), compatible with a more severe thromboinflammatory state, whereas counts in the persistent mild–moderate group remained within the normal range (≈ 234 × 10^9^/L). Given the small numbers, these differences likely reflect less pronounced endothelial/coagulation injury in persistent Stage 2 AKI rather than any protective effect of “higher” platelets. Given these considerations—and our small sample—we prioritized a simple, single‐draw composite, NLR/PLT, which places platelet count in the denominator and thus preserves the adverse information content of thrombocytopenia. Systemic immune‐inflammation index (SII = neutrophils × platelets ÷ lymphocytes) [25–28] was not associated with PS‐AKI and ranked low in SHAP importance—plausibly because placing platelets in the numerator may blunt the prognostic contribution of thrombocytopenia, a hallmark of persistent severe trajectories. In exploratory models, NLR/PLT was independently associated with PS‐AKI, and a two‐variable model (age + NLR/PLT) showed good apparent discrimination (AUC 0.86); internal cross‐validation yielded similar discrimination (AUC ≈ 0.79) but revealed limited calibration, and these findings require external validation. By contrast, procalcitonin was not associated with PS‐AKI in our data—plausibly reflecting its specificity for bacterial infection rather than global inflammatory tone—whereas CRP, NLR, and platelets may better capture the host inflammatory milieu that is theoretically more relevant to persistence than to the mere occurrence of AKI [29–31].

### 6.2. Clinical Implications

Although underpowered for definitive inference, this study adds data on the prevalence and outcome impact of PS‐AKI and suggests that accessible inflammatory–hematologic markers such as NLR, CRP, and platelets—summarized by NLR/PLT—may merit further investigation as exploratory early‐risk indicators. Future studies should test whether combining such simple indices with mechanistic biomarkers (e.g., CCL14, Ang‐2) can enhance prediction and prevention of PS‐AKI.

### 6.3. Limitations

This was a single‐center study with a modest sample size. Because the PS‐AKI definition was only recently standardized and no prior single‐center data from comparable settings were available, we used a fixed accrual window and consecutive sampling rather than a prespecified powered design. As a result, mortality effect estimates are imprecise, which we report with wide confidence intervals. Baseline creatinine assignment may still misclassify some patients; however, subgroup analyses restricted to those with outpatient baselines showed similar findings, with higher mortality and lower renal recovery in the PS‐AKI group (Supporting Table S13). Advanced biomarkers (e.g., CCL14, Ang‐2) were unavailable, and model performance from exploratory analyses may be optimistic given the small cohort and lack of external validation. In addition, the net fluid balance before AKI and prehospital nephrotoxic exposures were incompletely captured in this predominantly community‐acquired cohort, limiting our ability to fully characterize their relationship with AKI trajectories.

## 7. Conclusions

In this predominantly community‐acquired ICU cohort, PS‐AKI was frequent and carried the poorest prognosis, with low renal recovery and high in‐hospital mortality.

Elevated inflammatory markers together with thrombocytopenia—summarized by the NLR/PLT ratio—were observed among patients who developed PS‐AKI and merit prospective validation.

Overall, these findings contribute additional evidence on PS‐AKI and highlight the need for further research into early‐risk stratification and prevention of persistent severe trajectories [32].

**Nomenclature**
ADQI: Acute Disease Quality Initiative
AKI: Acute kidney injury
APACHE II: Acute Physiology and Chronic Health Evaluation II
AUC: Area under the receiver operating characteristic curve
CCI: Charlson Comorbidity Index
CKD: Chronic kidney disease
CRP: C‐reactive protein
eGFR: Estimated glomerular filtration rate
ICU: Intensive care unit
IRB: Institutional Review Board
KDIGO: Kidney Disease: Improving Global Outcomes
MDRD: Modification of Diet in Renal Disease
NLR: Neutrophil‐to‐lymphocyte ratio
NLR/PLT: Neutrophil‐to‐lymphocyte ratio divided by platelet count (× 100)
PCT: Procalcitonin
PS‐AKI: Persistent severe acute kidney injury
RRT: Renal replacement therapy
SII: Systemic immune‐inflammation index
SHAP: SHapley Additive exPlanations
SOFA: Sequential Organ Failure Assessment

## Author Contributions

• Tung Phi Nguyen: conceptualization; methodology; investigation; data curation; formal analysis; visualization; writing–original draft; writing–review and editing; project administration; and supervision (principal investigator).

• Thang Trong Khong: supervision; validation; resources; and writing–review and editing.

• Hoai Thi Thu Vu: supervision; validation; resources; and writing–review and editing.

• Nam Ngoc Phuong Nguyen: investigation; data curation; resources; and writing–review and editing.

• Phong Van Phan: investigation; data curation; resources; and writing–review and editing.

• Hue Thi Le: investigation; data curation; resources; and writing–review and editing.

• Tra Thi Hoang: investigation; data curation; resources; and writing–review and editing.

• Huyen Thi Nguyen: investigation; data curation; resources; and writing–review and editing.

• Loan Thi Phan: investigation; data curation; resources; and writing–review and editing.

• Yen Thi Kim Nguyen: investigation; data curation; resources; and writing–review and editing.

• Phuong Khanh Nguyen Hoang (Pharmacy Department): data curation (medication exposure extraction/validation); methodology (medication variable definitions); and writing–review and editing.

## Funding

This research received no specific grant from any funding agency, commercial, or not‐for‐profit sectors.

## Disclosure

All authors read and approved the final manuscript. This study was conducted as part of the authors’ employment at Vinmec International Hospital and VinUniversity.

## Ethics Statement

The study protocol was approved by the Institutional Review Board of VinUniversity with a waiver of informed consent, in accordance with the Declaration of Helsinki and applicable local regulations (IRB approval details available from the corresponding author upon request).

## Consent

The manuscript contains no individual person’s data in any form (including images).

## Conflicts of Interest

The authors declare no conflicts of interest.

## Supporting Information

Additional supporting information can be found online in the Supporting Information section.

## Supporting information


**Supporting Information 1** Supporting File S1. Ethics Approval (PDF)—Institutional Review Board approval document for the study (VinUniversity IRB).

**Supporting Information 2** Supporting File S2. Supporting Methods, Tables, and Figures (DOCX)—Detailed variable definitions (e.g., medication exposures), additional results (e.g., Tables S1–Sx), and sensitivity analyses/figures referenced in the text.

**Supporting Information 3** Supporting File S3. STROBE Checklist (DOCX)—Completed reporting checklist for cohort studies.

## Data Availability

The analysis code and deidentified datasets used in this study are available from the corresponding author upon reasonable request, subject to institutional approval and data‐sharing agreements. All potentially identifying patient information will be removed prior to data sharing to ensure confidentiality.
