# Supplementary material for: Persistent Severe Acute Kidney Injury Among Critically Ill Patients: Outcomes and Predictive Markers—A Single‐Center Retrospective Cohort Study
Source: Crit Care Res Pract. 2026 Feb 17;2026:6920702. doi: 10.1155/ccrp/6920702 (PMC12910387; doi:10.1155/ccrp/6920702)
Supplement: Supplementary file 1 — Supporting Information 1 Supporting File S1. Ethics Approval (PDF)—Institutional Review Board approval document for the study (VinUniversity IRB). [file CCRP-2026-6920702-s002.pdf]

CÔNG TY CỔ PHẦN BỆNH VIỆN ĐA KHOA QUỐC TẾ VINMEC
HỘI ĐỒNG ĐẠO ĐỨC TRONG NCYSH
Số: ..143.../2025/CN/HĐĐĐ VMEC
*V/v chấp thuận các vấn đề đạo đức NCYSH*

CỘNG HÒA XÃ HỘI CHỦ NGHĨA VIỆT NAM
**Độc lập - Tự do - Hạnh phúc**

*Hà Nội, ngày 20 tháng 8 năm 2025*

# GIẤY CHỨNG NHẬN

## CHẤP THUẬN CỦA HỘI ĐỒNG ĐẠO ĐỨC TRONG NGHIÊN CỨU Y SINH HỌC CÔNG TY CP BỆNH VIỆN ĐA KHOA QUỐC TẾ VINMEC – TRƯỜNG ĐẠI HỌC VINUNI

Căn cứ theo Thông tư 43/2024/TT-BYT quy định việc thành lập, tổ chức và hoạt động của Hội đồng đạo đức trong nghiên cứu y sinh học do Bộ trưởng Bộ Y tế ban hành ngày 12/12/2024

Căn cứ Quyết định số 24/2016/QĐ-VINMEC ngày 28/12/2016 của Chủ tịch Hội đồng quản trị Công ty Cổ phần Bệnh viện đa khoa quốc tế Vinmec về việc thành lập Hội đồng đạo đức trong Nghiên cứu y sinh học xét duyệt các vấn đề đạo đức trong nghiên cứu y sinh học của các đề tài/dự án;

Căn cứ Quyết định số 23/2016/QĐ-VINMEC ngày 28/12/2016 của Chủ tịch Hội đồng quản trị Công ty Cổ phần Bệnh viện đa khoa Quốc tế Vinmec về việc ban hành Điều lệ Tổ chức và hoạt động của Hội đồng đạo đức Công ty Cổ phần Bệnh viện đa khoa quốc tế Vinmec;

Căn cứ Quyết định số 524/2024/QĐ-VINMEC–03 ngày 23/12/2024 của Tổng Giám đốc Công ty Cổ phần Bệnh viện Đa khoa Quốc tế Vinmec về việc Kiện toàn Hội đồng đạo đức trong nghiên cứu Y, sinh học cấp công ty của Công ty Cổ phần Bệnh viện Đa khoa Quốc tế Vinmec – Trường Đại học VinUni.

Căn cứ Biên bản xét duyệt nghiên cứu ngày 20../..8../2025 của Hội đồng đạo đức trong nghiên cứu Y, sinh học cấp công ty của Công ty Cổ phần Bệnh viện Đa khoa Quốc tế Vinmec – Trường Đại học VinUni

1. Tên nghiên cứu: **Khảo sát tỷ lệ, yếu tố liên quan và kết cục của tổn thương thận cấp theo diễn tiến tại Khoa Hồi sức Bệnh viện Vinmec Central Park**
2. Nghiên cứu viên chính: BS. Nguyễn Phi Tùng, Khoa Hồi sức, Bệnh viện ĐKQT Vinmec Central Park
3. Đối tượng nghiên cứu: Tất cả bệnh nhân ≥ 18 tuổi được điều trị ≥ 24h tại khoa Hồi sức tích cực và có tổn thương thận cấp xác định theo tiêu chuẩn KDIGO 2012 trong thời gian nằm ICU trong giai đoạn từ 01/2024 – 06/2025
4. Cỡ mẫu: toàn bộ bệnh nhân AKI thỏa tiêu chí chọn mẫu trong khoảng thời gian 18 tháng của nghiên cứu

5. Địa điểm: Bệnh viện ĐKQT Vinmec Central Park

6. Thời gian thu thập dữ liệu: từ tháng 01/2024 đến tháng 06/2025

**Ngày chấp thuận:** 20 / 8 **/2025**

Những thay đổi trong khi sử dụng các tài liệu nêu trên sẽ phải được Hội đồng Đạo đức trong nghiên cứu y, sinh học cấp công ty của Công ty Cổ phần Bệnh viện đa khoa quốc tế Vinmec - Trường Đại học VinUni xem xét và chấp thuận trừ trường hợp phải thay đổi ngay để đảm bảo an toàn cao nhất cho đối tượng. Đồng thời phải báo cáo cho Hội đồng Đạo đức về những thay đổi này.

Nghiên cứu viên chính có trách nhiệm thực hiện đúng nguyên tắc về đạo đức đã được ghi trong đề cương nghiên cứu; các yêu cầu báo cáo về an toàn của Hội đồng Đạo đức và các quy định hiện hành về nghiên cứu lâm sàng đã được ban hành của Bộ Y tế; tuân thủ tuyên ngôn Helsinki và hướng dẫn thực hành lâm sàng tốt của Hội nghị Hợp nhất Quốc tế (ICH GCP).

***Nơi nhận:***

- *Nghiên cứu viên chính;*
- *Nhà tài trợ;*
- *Lưu: VT, P. QLNCKH;*

**CHỦ TỊCH HỘI ĐỒNG**

**GS.TS.BS. Đỗ Tất Cường**
