# Supplementary material for: Persistent Severe Acute Kidney Injury Among Critically Ill Patients: Outcomes and Predictive Markers—A Single‐Center Retrospective Cohort Study
Source: Crit Care Res Pract. 2026 Feb 17;2026:6920702. doi: 10.1155/ccrp/6920702 (PMC12910387; doi:10.1155/ccrp/6920702)
Supplement: Supplementary file 2 — Supporting Information 2 Supporting File S2. Supporting Methods, Tables, and Figures (DOCX)—Detailed variable definitions (e.g., medication exposures), additional results (e.g., Tables S1–Sx), and sensitivity analyses/figures referenced in the text. [file CCRP-2026-6920702-s003.docx]

Contents

[Supplementary Table S1. Nephrotoxic and diuretic agents screened in this study 2](#_Toc218109191)

[Supplementary Table S2: Pairwise log-rank comparisons and numbers-at-risk for Kaplan–Meier survival curves (Figure 2). 2](#_Toc218109192)

[Supplementary Table S3. Mortality models within KDIGO stage 2–3 (Cox) 3](#_Toc218109193)

[Supplementary Table S4. Renal-recovery models within KDIGO stage 2–3 (logistic) 3](#_Toc218109194)

[Supplementary Table S5. Association of PS-AKI with outcomes in the full cohort (N=106) 3](#_Toc218109195)

[Supplementary Table S6. Sensitivity analysis excluding early deaths (≤72 h) 4](#_Toc218109196)

[Supplementary Table S7. Landmark analysis at 72 h after first KDIGO stage 2–3 (Cox) 4](#_Toc218109197)

[Supplementary Table S8. Univariable predictors of PS-AKI (KDIGO stage 2–3) 5](#_Toc218109198)

[Supplementary Table S9. Two-variable models for PS-AKI (KDIGO stage 2–3) 5](#_Toc218109199)

[Supplementary Methods – Model performance assessment (TRIPOD reporting) 6](#_Toc218109200)

[Supplementary Results – Model performance 6](#_Toc218109201)

[Supplementary Table S10. Diagnostic performance of the age+NLR/PLT model at selected decision thresholds 6](#_Toc218109202)

[Supplementary Table S11. Median absolute SHAP values for predictors of PS-AKI (KDIGO stage 2–3) 7](#_Toc218109203)

[Supplementary Table S12. Median absolute SHAP values for predictors of PS-AKI (all AKI included) 7](#_Toc218109204)

[Supplementary Table S13. Characteristics and outcomes of patients with outpatient baseline creatinine (n = 28) 8](#_Toc218109205)

[Supplementary Table S14. AKI reversal phenotypes (early, late, never) across KDIGO stage 2–3 trajectories 8](#_Toc218109206)

[Supplementary Table S15. Hemodynamic profiles and vasopressor requirements prior to AKI diagnosis (worst values within 24 hours) 9](#_Toc218109207)

[Supplementary Figure S1. Kaplan–Meier survival from 72 h after first KDIGO stage 2–3 (PS-AKI vs non-PS-AKI), with numbers-at-risk 9](#_Toc218109208)

[Supplementary Figure S2. SHAP summary plot for predictors of PS-AKI (all AKI included). 10](#_Toc218109209)

# Supplementary Table S1. Nephrotoxic and diuretic agents screened in this study

| **Drug class** | **Agents (generic name)** |
| --- | --- |
| **Diuretics** | Furosemide, indapamide (thiazide-like), spironolactone (including fixed-dose combination with furosemide), acetazolamide |
| **NSAIDs** | Celecoxib, diclofenac, ibuprofen, indomethacin, ketoprofen, ketorolac, meloxicam, naproxen |
| **ACE inhibitors** | Captopril, enalapril, lisinopril, perindopril |
| **Angiotensin receptor blockers (ARBs)** | Irbesartan, losartan, olmesartan, telmisartan, valsartan |
| **Calcineurin inhibitors** | Cyclosporine, tacrolimus |
| **Anti-infectives (antibiotics / antivirals / antifungals)** | Acyclovir, adefovir, amikacin, amoxicillin, amphotericin B, benzathine, cefotaxime, ceftazidime, cefuroxime, chloroquine, ciprofloxacin, colistin, erythromycin, ganciclovir, gentamicin, levofloxacin, nafcillin, neomycin, nitrofurantoin, oseltamivir, oxacillin, piperacillin/tazobactam, polymyxin B, rifampin, streptomycin, sulfadiazine, tenofovir, ticarcillin, ticarcillin/clavulanate, tobramycin, trimethoprim/sulfamethoxazole, vancomycin |
| **Other nephrotoxins** | Allopurinol, cisplatin, colchicine, iodinated contrast media (iopromide, iobitridol), cyclophosphamide, intravenous immunoglobulin (IVIG), mannitol, mesalamine, methotrexate, phenytoin |

*Footnotes:* Presence of any listed diuretic or nephrotoxic agent during the pre-AKI window (from ICU admission up to—but not including—the calendar day of AKI diagnosis; for patients presenting with AKI on admission, the admission day was used) was coded as a binary exposure (Yes/No)

# Supplementary Table S2: Pairwise log-rank comparisons and numbers-at-risk for Kaplan–Meier survival curves (Figure 2).

| **Time (days)** | **Stage 1 AKI (n=45)** | **Non-persistent severe AKI (n=37)** | **Persistent severe AKI (n=24)** | **All groups (N=106)** |
| --- | --- | --- | --- | --- |
| 0 | 45 | 37 | 24 | 106 |
| 7 | 39 | 34 | 21 | 94 |
| 14 | 16 | 13 | 18 | 47 |
| 21 | 5 | 4 | 12 | 21 |
| 28 | 2 | 2 | 8 | 14 |
| 35 | 1 | 1 | 5 | 8 |
| 42 | 1 | 1 | 3 | 5 |
| 49 | 1 | 1 | 3 | 5 |
| 56 | 1 | 1 | 3 | 5 |

**Pairwise log-rank p-values (BH-adjusted):**

- Persistent severe AKI vs Non-persistent severe AKI: 0.145
- Persistent severe AKI vs Stage 1 AKI: 0.010
- Non-persistent severe AKI vs Stage 1 AKI: 0.145

**Footnote:** Log-rank tests compared overall survival between AKI trajectory groups. p-values were adjusted for multiple comparisons using the Benjamini–Hochberg method. Numbers-at-risk show the number of patients at risk (not censored or dead) at each time point from ICU admission**.**

# Supplementary Table S3. Mortality models within KDIGO stage 2–3 (Cox)

| **Model** | **N** | **Deaths** | **HR (95% CI)** | **p** |
| --- | --- | --- | --- | --- |
| Unadjusted | 61 | 17 | 2.59 (0.80–8.44) | 0.114 |
| Adjusted for non-renal SOFA (primary) | 61 | 17 | 2.23 (0.69–7.21) | 0.181 |
| Adjusted for age (per 10 y) | 61 | 17 | 2.65 (0.81–8.69) | 0.108 |
| Adjusted for CCI (per 1) | 61 | 17 | 2.04 (0.64–6.56) | 0.231 |
| Adjusted for SOFA at admission (per 1) | 61 | 17 | 1.73 (0.51–5.94) | 0.381 |
| Adjusted for APACHE II (per 5) | 61 | 17 | 2.24 (0.65–7.71) | 0.202 |

*Footnote:* HR, hazard ratio; CI, confidence interval; SOFA, Sequential Organ Failure Assessment; CCI, Charlson Comorbidity Index. Time origin was ICU admission; patients were censored at hospital discharge.

# Supplementary Table S4. Renal-recovery models within KDIGO stage 2–3 (logistic)

| **Model** | **N** | **Recovered n (%)** | **OR (95% CI)** | **p** |
| --- | --- | --- | --- | --- |
| Unadjusted | 61 | 32 (52.5) | 0.06 (0.02–0.22) | <0.001 |
| Adjusted for non-renal SOFA (primary) | 61 | 32 (52.5) | 0.07 (0.01–0.24) | <0.001 |
| Adjusted for age (per 10 y) | 61 | 32 (52.5) | 0.07 (0.02–0.24) | <0.001 |
| Adjusted for CCI (per 1) | 61 | 32 (52.5) | 0.09 (0.02–0.35) | 0.011 |
| Adjusted for SOFA at admission (per 1) | 61 | 32 (52.5) | 0.08 (0.02–0.28) | <0.001 |
| Adjusted for APACHE II (per 5) | 61 | 32 (52.5) | 0.10 (0.02–0.36) | <0.001 |

*Footnote:* OR, odds ratio; CI, confidence interval; SOFA, Sequential Organ Failure Assessment; CCI, Charlson Comorbidity Index. Renal recovery was defined as discharge sCr ≤150% of reference and no dialysis dependence.

# Supplementary Table S5. Association of PS-AKI with outcomes in the full cohort (N=106)

**A. In-hospital mortality (Cox)**

| **Model** | **N** | **Deaths** | **HR (95% CI)** | **p** |
| --- | --- | --- | --- | --- |
| Unadjusted | 106 | 18 | 4.42 (1.47–13.32) | 0.008 |
| Adjusted for non-renal SOFA (primary) | 106 | 18 | 3.12 (1.13–10.33) | 0.029 |
| Adjusted for age (per 10 y) | 106 | 18 | 4.44 (1.47–13.43) | 0.008 |
| Adjusted for CCI (per 1) | 106 | 18 | 3.02 (1.01–9.02) | 0.047 |
| Adjusted for SOFA at admission (per 1) | 106 | 18 | 2.77 (0.86–8.92) | 0.086 |
| Adjusted for APACHE II (per 5) | 106 | 18 | 2.70 (0.75–9.69) | 0.127 |

**B. Renal recovery (logistic)**

| **Model** | **N** | **Recovered n (%)** | **OR (95% CI)** | **p** |
| --- | --- | --- | --- | --- |
| Unadjusted | 106 | 70 (66.0) | 0.05 (0.01–0.15) | <0.001 |
| Adjusted for non-renal SOFA (primary) | 106 | 70 (66.0) | 0.06 (0.01–0.18) | <0.001 |
| Adjusted for age (per 10 y) | 106 | 70 (66.0) | 0.05 (0.01–0.15) | <0.001 |
| Adjusted for CCI (per 1) | 106 | 70 (66.0) | 0.07 (0.02–0.24) | <0.001 |
| Adjusted for SOFA at admission (per 1) | 106 | 70 (66.0) | 0.06 (0.02–0.20) | <0.001 |
| Adjusted for APACHE II (per 5) | 106 | 70 (66.0) | 0.09 (0.02–0.34) | <0.001 |

*Footnote:* HR, hazard ratio; OR, odds ratio; CI, confidence interval. Time origin was ICU admission; patients were censored at hospital discharge.

# Supplementary Table S6. Sensitivity analysis excluding early deaths (≤72 h)

| **Outcome** | **Non-PS-AKI (n=34)** | **PS-AKI  (n=23)** | **p-value** | **Adjusted effect*** |
| --- | --- | --- | --- | --- |
| In-hospital mortality,  n (%) | 3 (8.8) | 11 (47.8) | <0.01 | HR 2.18 (95% CI 0.64–7.46), p=0.21 |
| Renal recovery,  n (%) | 29 (85.3) | 4 (17.4) | <0.001 | OR 0.06 (95% CI 0.01–0.24), p<0.001 |

*Footnote:* *Adjusted for non-renal SOFA within 24 h of ICU admission. Time origin for survival was ICU admission; patients were censored at hospital discharge.*

# Supplementary Table S7. Landmark analysis at 72 h after first KDIGO stage 2–3 (Cox)

| **Model** | **n** | **Deaths** | **HR for PS-AKI  (95% CI)** | **p value** | **Covariates** | **Proportional hazards (global p)** |
| --- | --- | --- | --- | --- | --- | --- |
| **Unadjusted** | 50 | 10 | 4.14 (0.49–35.21) | 0.193 | PS-AKI only | 0.27 |
| **Parsimonious (primary)** | 50 | 10 | 4.13 (0.48–35.24) | 0.194 | PS-AKI +  non-renal SOFA | 0.25 |

Footnote: Time origin was 72 hours after the first KDIGO stage 2–3 episode. Patients who died or were discharged before the landmark (n = 6) were excluded from the risk set. Post-landmark deaths: 9/20 in PS-AKI vs 1/30 in non-PS-AKI; log-rank p = 0.20. Proportional hazards assumptions were met (global Schoenfeld p ≈ 0.25).

# Supplementary Table S8. Univariable predictors of PS-AKI (KDIGO stage 2–3)

| **Predictor** | **N** | **PS cases** | **OR (95% CI)** | **p** | **q (BH)** |
| --- | --- | --- | --- | --- | --- |
| NLR/PLT (per doubling) | 61 | 24 | 2.50 (1.53–4.06) | <0.001 | 0.0043 |
| NLR max (per doubling) | 61 | 24 | 2.89 (1.57–5.32) | <0.001 | 0.0064 |
| Platelet count min (per 10×10⁹/L) | 61 | 24 | 0.88 (0.82–0.96) | 0.0022 | 0.0119 |
| APACHE II (per 5) | 61 | 24 | 3.11 (1.49–6.48) | 0.0025 | 0.0119 |
| CRP max (per doubling) | 61 | 24 | 1.79 (1.20–2.68) | 0.0042 | 0.0158 |
| Charlson comorbidity index | 61 | 24 | 1.33 (1.08–1.64) | 0.0079 | 0.0251 |
| SOFA admission | 61 | 24 | 1.20 (1.03–1.39) | 0.0209 | 0.0568 |
| Any nephrotoxin before AKI | 61 | 24 | 4.67 (1.07–20.35) | 0.0403 | 0.0958 |
| BMI | 61 | 24 | 0.88 (0.77–1.01) | 0.0679 | 0.1210 |
| Lactate max (per doubling) | 58 | 24 | 1.71 (0.98–2.99) | 0.0592 | 0.1210 |
| Age | 61 | 24 | 1.02 (1.00–1.05) | 0.1098 | 0.1606 |
| Baseline creatinine | 61 | 24 | 1.01 (0.99–1.02) | 0.2303 | 0.2918 |
| SII (per doubling) | 61 | 24 | 1.27 (0.85–1.91) | 0.2468 | 0.2931 |
| Any diuretics before AKI | 61 | 24 | 2.25 (0.54–9.34) | 0.2643 | 0.2954 |
| Procalcitonin max (per doubling) | 57 | 24 | 1.10 (0.87–1.38) | 0.4234 | 0.4470 |

**Footnote:** Continuous predictors were log₂-transformed where modeled “per doubling” (e.g., NLR/PLT, NLR, CRP). Platelet OR is per 10×10⁹/L; APACHE II per 5 points. p-values are from logistic regression; q-values are Benjamini–Hochberg adjusted.

# Supplementary Table S9. Two-variable models for PS-AKI (KDIGO stage 2–3)

| **Model** | **N** | **PS cases** | **AUC (95% CI)** | **Term** | **OR (95% CI)** | **p** |
| --- | --- | --- | --- | --- | --- | --- |
| age + NLR/PLT (primary) | 61 | 24 | 0.86 (0.76–0.96) | NLR/PLT (per doubling) | 2.51 (1.52–4.12) | <0.001 |
| age + NLR max | 61 | 24 | 0.81 (0.70–0.92) | NLR max (per doubling) | 2.86 (1.55–5.30) | <0.001 |
| age + PLT min | 61 | 24 | 0.79 (0.67–0.92) | PLT min (per 10×10⁹/L) | 0.89 (0.82–0.96) | 0.0027 |
| age + CRP max | 61 | 24 | 0.80 (0.69–0.92) | CRP max (per doubling) | 1.84 (1.20–2.82) | 0.0051 |
| first AKI stage + NLR/PLT | 61 | 24 | 0.88 (0.79–0.96) | NLR/PLT (per doubling) | 2.19 (1.32–3.65) | 0.0025 |
| APACHE II + NLR/PLT | 61 | 24 | 0.87 (0.78–0.96) | NLR/PLT (per doubling) | 2.19 (1.32–3.62) | 0.0022 |

**Footnote:** Logistic regression models with two predictors. AUC values are from 5-fold cross-validation. The primary prespecified model was age + NLR/PLT.

## Supplementary Methods – Model performance assessment (TRIPOD reporting)

To evaluate the performance of the prespecified two-variable model (age + NLR/PLT) for predicting PS-AKI, we performed 5-fold stratified cross-validation. Discrimination was assessed by area under the receiver operating characteristic curve (AUC) with 95% confidence intervals (DeLong’s method). Calibration-in-the-large (intercept) was estimated from a logistic model with offset logit(p̂), and calibration slope from a logistic model regressing outcomes on logit(p̂). Calibration was summarized by calibration-in-the-large (intercept) and calibration slope with 95% CIs.
Two decision thresholds were examined: the Youden-optimal threshold and the threshold achieving the highest possible sensitivity (target ≈0.90). For each threshold, sensitivity, specificity, positive predictive value (PPV), and negative predictive value (NPV) were reported with exact 95% binomial confidence intervals**.**

## Supplementary Results – Model performance

The cross-validated AUC was 0.79 (95% CI 0.67–0.91). Calibration-in-the-large was 0.03 (95% CI −0.54 to 0.58) and the calibration slope 0.14 (95% CI −0.06 to 0.52), indicating well-centered but under-dispersed predictions.
At the Youden threshold (0.40), sensitivity was 0.71 (95% CI 0.49–0.87) and specificity 0.78 (95% CI 0.62–0.90).
At the high-sensitivity threshold (0.28), sensitivity was 0.92 (95% CI 0.73–0.99) and specificity 0.59 (95% CI 0.42–0.74).
Full decision threshold metrics are presented in Supplementary Table S10.

# Supplementary Table S10. Diagnostic performance of the age+NLR/PLT model at selected decision thresholds

| **Decision rule** | **Probability threshold** | **Sensitivity  (95% CI)** | **Specificity  (95% CI)** | **Positive predictive value (PPV, 95% CI)** | **Negative predictive  value (NPV, 95% CI)** |
| --- | --- | --- | --- | --- | --- |
| Youden-optimal | 0.396 | 0.71 (0.49–0.87) | 0.78 (0.62–0.90) | 0.68 (0.46–0.85) | 0.81 (0.64–0.92) |
| High-sensitivity target | 0.282 | 0.92 (0.73–0.99) | 0.59 (0.42–0.74) | 0.50 (0.35–0.65) | 0.88 (0.64–0.99) |

**Footnote:**Predicted probabilities were obtained from 5-fold stratified cross-validation of the prespecified two-variable logistic regression model (age + NLR-to-platelet ratio). Sensitivity = TP / (TP + FN); Specificity = TN / (TN + FP); PPV = TP / (TP + FP); NPV = TN / (TN + FN). 95% confidence intervals were calculated using the exact binomial method.
The Youden-optimal threshold was chosen to maximize (sensitivity + specificity − 1). The high-sensitivity threshold was the lowest threshold achieving the maximum possible sensitivity (target ≈0.90) while maintaining acceptable specificity**.**

# Supplementary Table S11. Median absolute SHAP values for predictors of PS-AKI (KDIGO stage 2–3)

| **Predictor** | **Median \|SHAP\|** | **Note** |
| --- | --- | --- |
| Max NLR (log₂) | 0.661 | Per doubling |
| Max CRP (log₂) | 0.455 | Per doubling |
| Min platelets | 0.415 | ×10⁹/L |
| NLR/PLT (log₂) | 0.404 | Per doubling |
| Age | 0.321 | Per year |
| APACHE II | 0.279 | Per 5 points |
| Norepinephrine dose (log₂) | 0.278 | — |
| Baseline creatinine | 0.274 | µmol/L |
| Max lactate (log₂) | 0.261 | Per doubling |
| First AKI stage | 0.182 | 3 vs 2 |
| Charlson index | 0.094 | Per 1 point |
| SII (log₂) | 0.077 | Per doubling |
| Nephrotoxin count | 0.035 | Number |
| SOFA at admission | 0.028 | Points |

*Footnote:* Features are ranked by median absolute SHAP values from the gradient-boosted model (N = 61; KDIGO stage 2–3). Log₂-transformed predictors are interpreted “per doubling.” AUC = 0.842 (95% CI 0.74–0.93).

# Supplementary Table S12. Median absolute SHAP values for predictors of PS-AKI (all AKI included)

| **Predictor** | **Median \|SHAP\|** | **Note** |
| --- | --- | --- |
| APACHE II (per 5) | 0.793 | Per 5 points |
| First AKI stage (1–3) | 0.529 | 1–3 ordinal |
| NLR/PLT (log₂ per doubling) | 0.315 | Per doubling |
| Max NLR (log₂ per doubling) | 0.269 | Per doubling |
| BMI (per 1 unit) | 0.242 | kg/m² |
| Max CRP (log₂ per doubling) | 0.232 | Per doubling |
| Max lactate (log₂ per doubling) | 0.199 | Per doubling |
| Norepinephrine dose (log₂) | 0.141 | — |
| Min platelets (10⁹/L) | 0.138 | ×10⁹/L |
| Baseline creatinine (raw) | 0.134 | µmol/L |
| Charlson index (per 1) | 0.073 | Per 1 point |
| Age (per 1 year) | 0.036 | Per year |
| SII (log₂) | 0.032 | Per doubling |
| SOFA non-renal (per 1) | 0.031 | Points |
| SOFA at admission (per 1) | 0.019 | Points |

*Footnote:* Features are ranked by median absolute SHAP values from the gradient-boosted model (N = 106; all AKI included). Log₂-transformed predictors are interpreted “per doubling.” AUC = 0.916 (95% CI 0.86–0.97). Supplementary Figure S2 shows the corresponding bee-swarm plot.

# Supplementary Table S13. Characteristics and outcomes of patients with outpatient baseline creatinine (n = 28)

This subgroup analysis was restricted to patients with a documented outpatient baseline serum creatinine (sCr), to mitigate potential misclassification related to baseline assignment. Results were consistent with the main analysis, with markedly higher mortality and lower renal recovery in the PS-AKI group.

**(A) Baseline characteristics (KDIGO 2–3)**

| **Group** | **N** | **Age (years)** | **APACHE II** | **Non-renal SOFA** |
| --- | --- | --- | --- | --- |
| PS-AKI | 14 | 84.0 (64.5–91.2) | 31.0 (28.2–32.5) | 5.0 (3.0–6.8) |
| Non-PS | 14 | 73.5 (51.0–87.2) | 27.0 (25.0–30.8) | 4.5 (3.0–6.8) |

**(B) Outcomes (KDIGO 2–3)**

| **Group** | **N** | **Deaths, n (%)** | **Renal recovery, n/N (%)** |
| --- | --- | --- | --- |
| Non-PS | 14 | 3 (21.4%) | 9/14 (64.3%) |
| PS-AKI | 14 | 8 (57.1%) | 1/14 (7.1%) |

**(C) Mortality HR (outpatient-baseline cohort)***Model not estimable due to missing time-to-event data; mortality proportions are shown in panel B.*

**(D) Renal recovery OR (KDIGO 2–3)**

| **Model** | **N** | **Recovered, n (%)** | **OR** | **95% CI** | **p** |
| --- | --- | --- | --- | --- | --- |
| Unadjusted (PS-AKI → recovery) | 28 | 10 (35.7%) | 0.04 | 0.00–0.31 | 0.007 |
| Adjusted for non-renal SOFA | 28 | 10 (35.7%) | 0.03 | 0.00–0.23 | 0.005 |

# Supplementary Table S14. AKI reversal phenotypes (early, late, never) across KDIGO stage 2–3 trajectories

| **Reversal phenotype** | **Transient AKI (n=23)** | **Persistent mild–moderate AKI (n=14)** | **Persistent severe AKI (n=24)** | ***p*-value** |
| --- | --- | --- | --- | --- |
| **Early reversal (≤7 days)** | 23 (100) | 2 (14.3) | 2 (8.3) | <0.01 |
| **Late reversal (>7 days)** | 0 (0) | 5 (35.7) | 2 (8.3) | <0.01 |

**Footnotes:**
Reversal definitions followed Kellum *et al.*: **early reversal ≤7 days**, **late reversal >7 days**, **never reversed = no recovery before discharge/death**.

# Supplementary Table S15. Hemodynamic profiles and vasopressor requirements prior to AKI diagnosis (worst values within 24 hours)

| **Characteristic** | **Transient AKI (n=23)** | **Persistent mild–moderate (n=14)** | **Persistent severe (n=24)** | **p-value** |
| --- | --- | --- | --- | --- |
| Mean Arterial Pressure (MAP) at admission, mmHg | 72 (66–80) | 69 (63–75) | 65 (59–70) | 0.02 |
| Norepinephrine equivalent dose at admission, µg/kg/min | 0.00 (0.00, 0.05) | 0.00 (0.00, 0.06) | 0.09 (0.00, 0.21) | < 0.001 |

**Footnotes:** Data reflect hemodynamic parameters recorded at the time of ICU admission.

- **MAP at admission:** The initial mean arterial pressure recorded upon ICU entry. **Norepinephrine dose:** The infusion rate required at the time of admission to maintain target blood pressure.
- Since 91.5% of the cohort presented with community-acquired AKI (diagnosed at or shortly after admission), these admission values serve as a proxy for the hemodynamic status at AKI onset

# Supplementary Figure S1. Kaplan–Meier survival from 72 h after first KDIGO stage 2–3 (PS-AKI vs non-PS-AKI), with numbers-at-risk


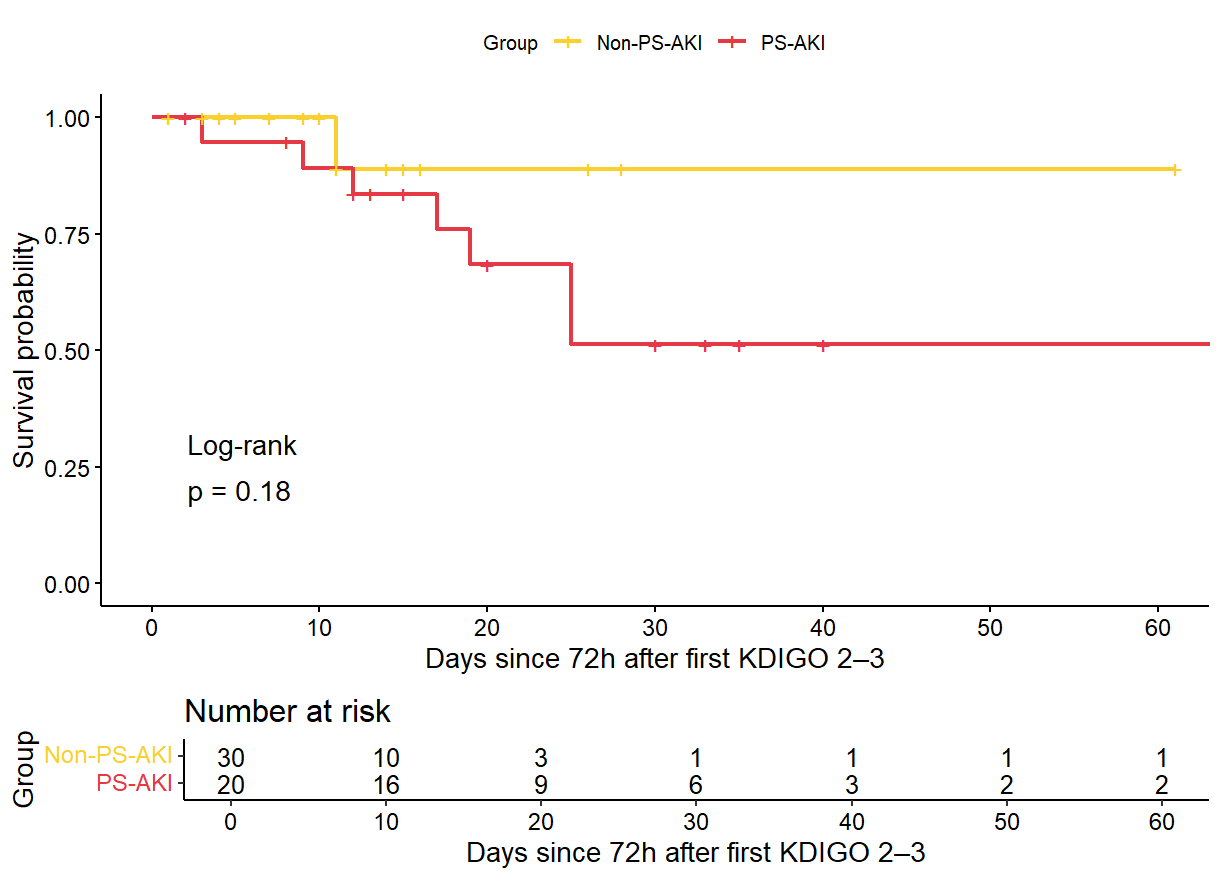


Kaplan–Meier survival from 72 h after first KDIGO stage 2–3 (landmark analysis). Numbers-at-risk are shown below the x-axis. Post-landmark deaths were 9/20 in PS-AKI vs 1/30 in non-PS-AKI; log-rank p=0.20

# Supplementary Figure S2. SHAP summary plot for predictors of PS-AKI (all AKI included).


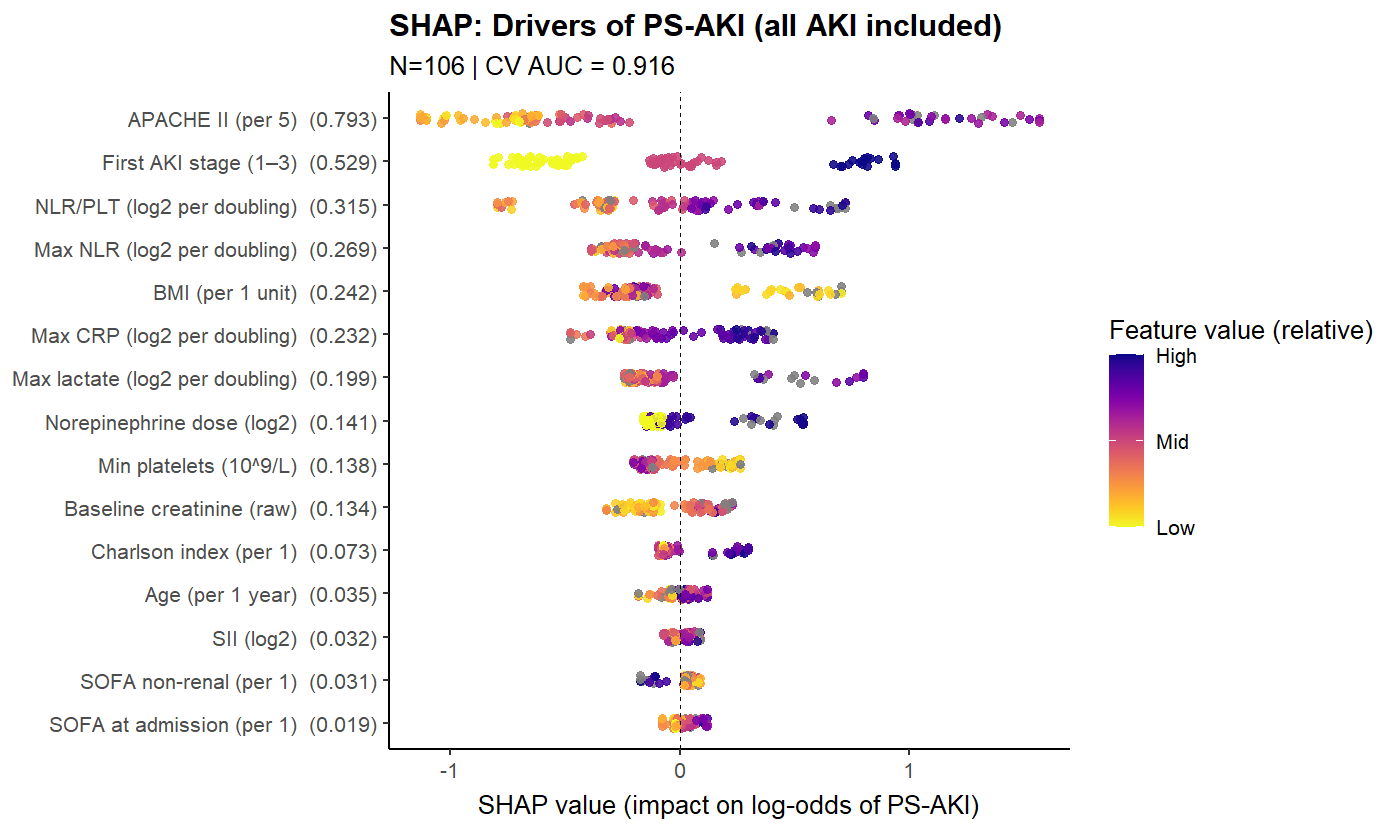


*Legend:* Bee-swarm plot from the gradient-boosted model including all AKI patients (N = 106; 5-fold CV AUC = 0.916). Features are ordered by median |SHAP|; point color encodes relative feature value (feature-wise min–max normalization).
