# Supplementary material for: Persistent Severe Acute Kidney Injury Among Critically Ill Patients: Outcomes and Predictive Markers—A Single‐Center Retrospective Cohort Study
Source: Crit Care Res Pract. 2026 Feb 17;2026:6920702. doi: 10.1155/ccrp/6920702 (PMC12910387; doi:10.1155/ccrp/6920702)
Supplement: Supplementary file 3 — Supporting Information 3 Supporting File S3. STROBE Checklist (DOCX)—Completed reporting checklist for cohort studies. [file CCRP-2026-6920702-s001.docx]

# STROBE Statement—Checklist for Cohort Studies (Completed for: PS‑AKI manuscript)

Notes: Page numbers vary by layout; locations are given by section names and anchor phrases so editors can verify rapidly. Items marked “ADD” need minor edits before submission.

| **Item** | **Recommendation (short)** | **Where/How addressed in manuscript** | **Notes / Action** |
| --- | --- | --- | --- |
| **1a** | Study design in title/abstract | Abstract states 'retrospective cohort'. Title: ensure design term included. | OK |
| **1b** | Informative abstract | Structured abstract summarises setting, participants (N=106), outcomes (mortality, renal recovery), and key estimates. | OK (≤300 words). |
| **2** | Background/rationale | Intro explains PS‑AKI clinical impact and evidence gap. | OK |
| **3** | Objectives | States aims: classify AKI trajectories, evaluate outcomes, explore predictors (age+NLR/PLT). | OK |
| **4** | Key elements of study design early | Methods → 'Study design and population': single‑centre retrospective cohort at tertiary hospital, consecutive adults. | OK |
| **5** | Setting, locations, dates | Vietnam tertiary academic hospital affiliated with VinUniversity; admissions Jan 2024–Jun 2025; data window defined. | OK |
| **6a** | Eligibility & selection; follow‑up | KDIGO‑based AKI; inclusion/exclusion fully listed; follow‑up to hospital discharge. | OK |
| **6b** | Matching (if any) | Not a matched study. | N/A |
| **7** | Clearly define variables | Outcomes: in‑hospital mortality (primary), renal recovery; Trajectories per Gómez; Predictors include NLR/PLT, etc. | OK |
| **8*** | Data sources/measurement | Data abstracted from EHR; biomarker windows and computations (e.g., NLR/PLT ×100, SII) specified. | OK |
| **9** | Bias | Reference creatinine hierarchy to mitigate misclassification; confounding controlled by non‑renal SOFA; sensitivity analyses (landmark, exclude early deaths). | OK |
| **10** | Study size | Consecutive convenience sample over study period; no prior sample‑size calculation. | OK |
| **11** | Handling quantitative variables | Skewed biomarkers analysed on log2 (per doubling); platelets per 10×10⁹/L; models specified. | OK |
| **12a** | Statistical methods incl. confounding | KM/log‑rank; Cox adjusted for non‑renal SOFA; logistic for renal recovery; prespecified age+NLR/PLT model; XGBoost+SHAP. | OK |
| **12b** | Subgroups/interactions | Exploratory predictor models across KDIGO stage 2–3 and all AKI. | OK/Exploratory |
| **12c** | Missing data | Not fully explicit; appears complete‑case for biomarkers with varying N. | OK |
| **12d** | Loss to follow‑up | In‑hospital cohort; censor at discharge. | OK/Not applicable. |
| **12e** | Sensitivity analyses | Landmark at 72h; exclude early deaths; outpatient baseline sCr subgroup. | OK |
| **13a*** | Participant numbers at each stage | 164 screened → 106 included; groups detailed; flow diagram (Fig 1). | OK |
| **13b*** | Reasons for non‑participation | Exclusions: ESKD/Tx, extreme baseline sCr/eGFR without evidence, insufficient data, age <18. | OK |
| **13c*** | Flow diagram | Figure 1 provided. | OK |
| **14a*** | Descriptive data | Table 1: demographics, severity, exposures and confounders by group. | OK |
| **14b*** | Missing data by variable | Partially indicated via differing Ns (e.g., PCT). | OK |
| **14c*** | Follow‑up time | ICU and hospital length of stay reported by group. | OK |
| **15*** | Outcome events over time | Mortality and renal‑recovery counts by group; KM curves with numbers‑at‑risk (Supplementary S2). | OK |
| **16a** | Main results: estimates, precision, adjustments | Adjusted HR/OR with 95% CI (main text & Supplementary S3–S5). | OK |
| **16b** | Category boundaries | Continuous variables mostly kept continuous; when categorized (e.g., thresholds), boundaries shown in tables/footnotes. | OK |
| **16c** | Translate to absolute risk (if relevant) | Absolute risks by group are reported (e.g., mortality %). | OK |
| **17** | Other analyses | Sensitivity analyses; model performance (AUC, calibration); SHAP importance. | OK |
| **18** | Key results in Discussion | Discussion summarises objectives and key findings. | OK |
| **19** | Limitations | Single centre; modest N; residual confounding; baseline creatinine misclassification; biomarker availability. | OK |
| **20** | Interpretation | Cautious interpretation consistent with data and literature; need external validation. | OK |
| **21** | Generalisability | Discussed in context of single‑centre, community‑acquired cohort in Vietnam. | OK |
| **22** | Funding & role of funders | No external funding; institutional employment only. | OK |
